# Mirtazapine added to SSRIs or SNRIs for treatment resistant depression in primary care: phase III randomised placebo controlled trial (MIR)

**DOI:** 10.1136/bmj.k4218

**Published:** 2018-10-31

**Authors:** David S Kessler, Stephanie J MacNeill, Deborah Tallon, Glyn Lewis, Tim J Peters, William Hollingworth, Jeff Round, Alison Burns, Carolyn A Chew-Graham, Ian M Anderson, Tom Shepherd, John Campbell, Chris M Dickens, Mary Carter, Caroline Jenkinson, Una Macleod, Helen Gibson, Simon Davies, Nicola J Wiles

**Affiliations:** 1 Population Health Sciences, Bristol Medical School, Bristol BS8 2BN, UK; 2 Bristol Randomised Trials Collaboration, Population Health Sciences, Bristol Medical School, Bristol, UK; 3 Division of Psychiatry, University College London, London, UK; 4 Primary Care and Health Sciences, Keele University, Keele, UK; 5 Division of Neuroscience and Experimental Psychology, University of Manchester, Manchester, UK; 6 Exeter Medical School, University of Exeter, Exeter, UK; 7 Hull York Medical School, Hull, UK; 8 Centre for Addiction and Mental Health, Toronto, Canada

## Abstract

**Objective:**

To investigate the effectiveness of combining mirtazapine with serotonin-noradrenaline reuptake inhibitor (SNRI) or selective serotonin reuptake inhibitor (SSRI) antidepressants for treatment resistant depression in primary care.

**Design:**

Two parallel group multicentre phase III randomised placebo controlled trial.

**Setting:**

106 general practices in four UK sites; Bristol, Exeter, Hull, and Keele/North Staffs, August 2013 to October 2015.

**Participants:**

480 adults aged 18 or more years who scored 14 or more on the Beck depression inventory, second revision, fulfilled ICD-10 (international classification of diseases, 10th revision) criteria for depression, and had used an SSRI or SNRI for at least six weeks but were still depressed. 241 were randomised to mirtazapine and 239 to placebo, both given in addition to usual SSRI or SNRI treatment. Participants were stratified by centre and minimised by baseline Beck depression inventory score, sex, and current psychological therapy. They were followed up at 12, 24, and 52 weeks. 431 (89.8%) were included in the (primary) 12 week follow-up.

**Main outcome measures:**

Depressive symptoms at 12 weeks after randomisation, measured using the Beck depression inventory II score as a continuous variable. Secondary outcomes included measures of anxiety, quality of life, and adverse effects at 12, 24, and 52 weeks.

**Results:**

Beck depression inventory II scores at 12 weeks were lower in the mirtazapine group after adjustment for baseline scores and minimisation or stratification variables, although the confidence interval included the null (mean (SD) scores at 12 weeks: 18.0 (12.3) in the mirtazapine group, 19.7 (12.4) in the placebo group; adjusted difference between means −1.83 (95% confidence interval −3.92 to 0.27); P=0.09). Adverse effects were more common in the mirtazapine group and were associated with the participants stopping the trial drug.

**Conclusion:**

This study did not find evidence of a clinically important benefit for mirtazapine in addition to an SSRI or SNRI over placebo in a treatment resistant group of primary care patients with depression. This remains an area of important unmet need where evidence of effective treatment options is limited.

**Trial registration:**

Current Controlled Trials ISRCTN06653773.

**Figure:**
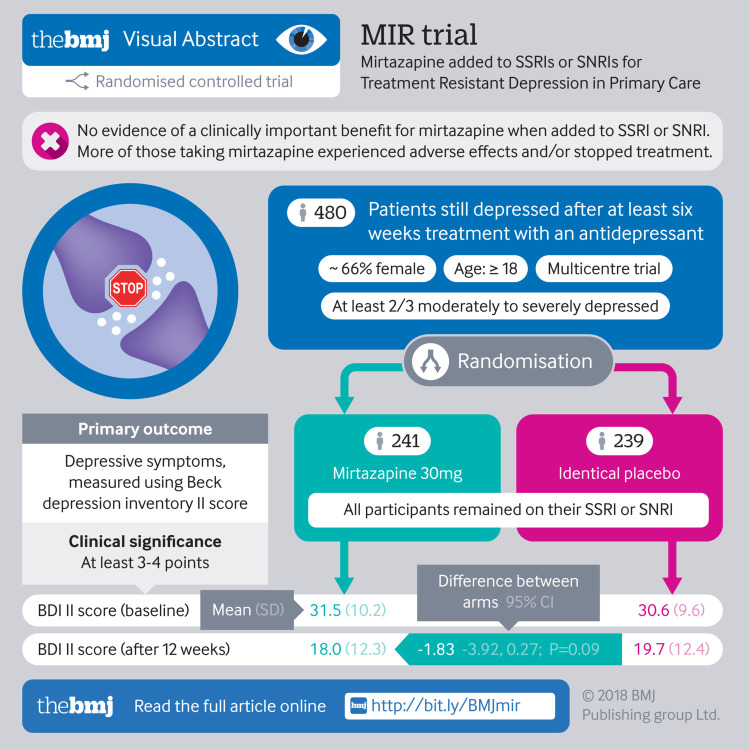


## Introduction

Depression is among the top five contributors to the global burden of disease, and by 2030 is predicted to be the leading cause of disability in high income countries.^1^ People with depression in the United Kingdom are usually managed in primary care, and antidepressants are often the first line treatment. The number of prescriptions for antidepressants has risen dramatically in recent years in the National Health Service, increasing by 6.8% (3.9 million items) during 2014-15 (total 61 million items).^2^ Many patients, however, do not respond to treatment. The STAR*D study (Sequenced Treatment Alternatives to Relieve Depression) found that half of those treated failed to experience at least a 50% reduction in depressive symptoms after 12-14 weeks of treatment with a single antidepressant.^3^ A substantial proportion of those who take antidepressants in an adequate dose and for an adequate period do not experience a clinically meaningful improvement in depressive symptoms.^3^

The National Institute for Health and Care Excellence advises general practitioners to reconsider treatment if patients show no response after 4-6 weeks of antidepressant use.^4^ Limited evidence is currently available to guide doctors in the management of patients who meet the ICD-10 (international classification of diseases, 10th revision) criteria for depression after taking a serotonin-noradrenaline reuptake inhibitor (SNRI) or selective serotonin reuptake inhibitor (SSRI) at an adequate dose for a minimum of six weeks.^5^ Several drug strategies have been proposed, including increasing the dose, switching antidepressants, combining two antidepressants, and augmenting the antidepressant with another psychotropic drug, such as lithium or an antipsychotic.^6^ A systematic review of antidepressant combinations for those who did not respond to monotherapy found that the small number of trials and methodological drawbacks of those trials precluded definitive conclusions about effectiveness, and some of the combinations carry a substantial risk of adverse effects and are not considered appropriate for initiation in primary care.^7^ There is a pharmacological rationale for adding a second antidepressant with a different and complementary mode of action to SSRIs or SNRIs. Mirtazapine, a noradrenaline (α2 adrenoreceptor) and serotonin (5 hydroxtryptamine receptors 2 and 3) antagonist, has the potential for an additive and perhaps synergistic action with SSRIs and SNRIs and could enhance clinical response compared with monotherapy with SSRIs or SNRIs. Four trials have been carried out of this combination against SSRI and SNRI monotherapy in participants who are treatment resistant and in those without treatment failure, with mixed results.^8^ ^9^ ^10^ ^11^

We determined the effectiveness of adding mirtazapine to an SSRI or SNRI in reducing depressive symptoms and improving quality of life at 12 weeks (primary follow-up) and at 24 and 52 weeks compared with adding placebo for patients in primary care who still experience depression after an adequate course of treatment.

## Methods

### Study design and participants

The MIR Study was a two parallel group multicentre pragmatic placebo controlled randomised trial with allocation at the level of the individual. We recruited participants from general practices in areas surrounding the four centres of Bristol, Exeter, Hull, and Keele/North Staffs. Eligible participants were aged 18 years or more, had used an SSRI or SNRI antidepressant at an adequate dose for at least six weeks, were adherent to treatment, had a Beck depression inventory, second revision (BDI II) score of 14 or more,^12^ and fulfilled the ICD-10 criteria for depression. We excluded patients with bipolar disorder, psychosis, major alcohol or substance misuse, a diagnosis of dementia, and an inability to complete the questionnaires, and women who were pregnant, breast feeding, or planning pregnancy.

We used a three stage recruitment process to identify potential participants. Staff at general practices searched their computerised records to identify patients who had received repeated prescriptions for an antidepressant during the previous four months and were being prescribed an antidepressant at an adequate dose. Doctors screened this list of patients and excluded those on the basis of the study eligibility criteria. Potentially eligible participants received a letter of invitation and brief information about the study, seeking permission for the research team to contact them. Doctors could also invite patients during a consultation to take part in the study, in which case the doctor provided information about the study and obtained permission to pass contact details to the research team. Those who agreed to be contacted were sent a postal questionnaire. This included questions about their depressive symptoms (BDI II) and use of antidepressants.

To ascertain eligibility a researcher telephoned those who met the initial criteria of severity of depressive symptoms and adherence to an adequate dose of an antidepressant for at least six weeks. Face to face baseline assessments were then conducted in the participants’ own homes, at their general practices, or at nearby National Health Service or university premises. Only those patients who fulfilled ICD-10 criteria (category F32) for their current depressive episode (assessed using the revised clinical interview schedule),^13^ had a BDI II score of 14 or more and who were continuing to take the prescribed antidepressants at an adequate dose were eligible to participate in the trial.

### Randomisation and masking

Those who were eligible and gave written informed consent were randomised to either one 15 mg capsule of mirtazapine daily for two weeks followed by two 15 mg capsules of mirtazapine for up to 50 weeks, or to identical placebo.

Randomisation was by means of a computer generated code, ensuring that allocation was concealed from the recruiting researcher. Randomisation was stratified by centre and minimised on baseline BDI II score (mild <26, moderate 26-34, or severe ≥35), sex (men or women), and current receipt of psychological services (yes or no).

The Medicines and Healthcare products Regulatory Authority approved the labelling of the drug packs. Each pack had an identification number, randomly generated to ensure that mirtazapine and placebo packs were indistinguishable to maintain allocation concealment. The Bristol Randomised Trials Collaboration generated the random numbers for the manufacturer. Participants and doctors were advised to use other serotonergic drugs with caution, such as tramadol or the triptan group of drugs.

Participants were free to stop taking the study drug at any time. Participants, clinicians, outcome assessors, and the research team were blinded to allocation. After the primary follow-up at 12 weeks, participants were offered the opportunity to be unblinded. This was not in the original protocol but was required by the research ethics committee to ensure that those who had not improved had the option of reviewing their treatment. Those who elected to be unblinded no longer received the trial drug, but outcome measures continued to be collected. Participants continued with care through their doctor and usual antidepressants. Clinicians were not restricted in referring their patients to psychological services.

### Procedures

Participants were followed up at 6, 12, 24, and 52 weeks. To maximise response rates, follow-up assessments at 12, 24, and 52 weeks were conducted at a face-to-face appointment with a researcher. If this was not possible then questionnaires were posted or administered over the phone.

The primary outcome was BDI II score at 12 weeks after randomisation, measured as a continuous variable, adjusted for baseline. We aimed to recruit 200 participants in each group, giving 91% power to detect a difference of 0.33 standard deviations at a two sided 5% significance level. This would be equivalent to a between group difference of 3 or 4 points on the BDI II, reported to be a clinically important difference.^14^ Allowing for 15% loss to follow-up at 12 weeks, we planned to recruit 472 participants.

Secondary outcomes were: response, defined as at least a 50% reduction in BDI II score compared with baseline; remission, defined as a score of less than 10 on the BDI II; depression using the patient health questionnaire (PHQ-9),^15^ a brief measure included because it is widely used in primary care: anxiety symptoms measured with the generalised anxiety disorder (GAD-7)^16^ assessment; adverse effects using the antidepressant side effect checklist^17^; quality of life measured using the EQ-5D-5L^18^; social and physical functioning using SF-12^19^; and adherence to antidepressants using a four item self report measure.^20^ Secondary outcomes were measured at 12, 24 (excluding antidepressant side effect checklist), and 52 weeks, with adjustments for baseline scores where appropriate. Cost effectiveness data will be presented in a separate publication.

### Statistical analysis

Analysis and reporting were in line with CONSORT^21^ guidelines based on a prespecified statistical analysis plan approved by the trial steering committee.^22^ Primary analyses compared the two groups as randomised, without imputing missing values. Depending on the type of outcome variable (continuous or binary), we used linear or logistic regression models to compare the groups as randomised, adjusting for stratification and minimisation variables and (where available) the corresponding baseline value.

Secondary analyses of the primary and secondary outcomes included additional adjustment for variables showing noticeable imbalance at baseline (ascertained using descriptive statistics).

In the analyses, we present regression coefficients (or odds ratios for binary outcomes), with 95% confidence intervals and P values. Effect sizes are presented for the BDI II outcomes and are calculated based on Cohen’s d statistic.

In prespecified subgroup analyses we introduced appropriate interaction terms into the regression models to investigate differential effects according to baseline severity of depression (BDI II), and we carried out a multilevel measure of degree of treatment resistance based on duration of symptoms and previous treatment with antidepressants. This latter variable was categorised as: not prescribed antidepressants in the past; prescribed antidepressants in the past, and depressed for less than one year; prescribed antidepressants in the past and depressed for one or two years; and prescribed antidepressants in the past and depressed for more than two years.

To assess the robustness of our primary analysis, we carried out sensitivity analyses. These included per protocol analyses of the primary outcome at 12 and 52 weeks and, since these were likely to be biased, a complier average causal effect analysis at 12, 24, and 52 weeks.^23^ In this analysis we defined participants who adhered to treatment as those who had continued taking their trial drug up until 12 weeks. An additional sensitivity analysis at 24 and 52 weeks examined between group differences in BDI II score in those who remained blinded throughout the trial. We also investigated the influence of missing data by performing analyses of the primary outcome under different assumptions: “best” and “worst” case scenarios (representing the lowest and highest possible BDI II scores) and multiple imputation by chained equation to impute missing data.^24^ When using this equation, we generated 25 datasets, and we undertook 10 switching procedures. The imputation model included all variables predictive of missingness as well as variables used in the primary analysis.

Analyses were performed using Stata v14.^25^

### Patient and public involvement

Patient and service user groups from Bristol and Manchester (PRIMER) were involved in the development of the full application and commented on the plain English summary. All of them said that they recognised the value of the trial and offered advice about recruitment strategies. The Research Materials Advisory Service of the West Hub Mental Health Research Network (now Clinical Research Network) worked with the trial team to develop patient information materials and consent forms. A panel of service users reviewed the study documents before they were sent for ethical approval. A patient representative sat on the trial steering committee. A patient group met regularly to contribute to the nested qualitative study; this group advised on topic guides, contributed to analysis of the qualitative datasets, and advised on dissemination activities.

## Results

**Fig 1:**
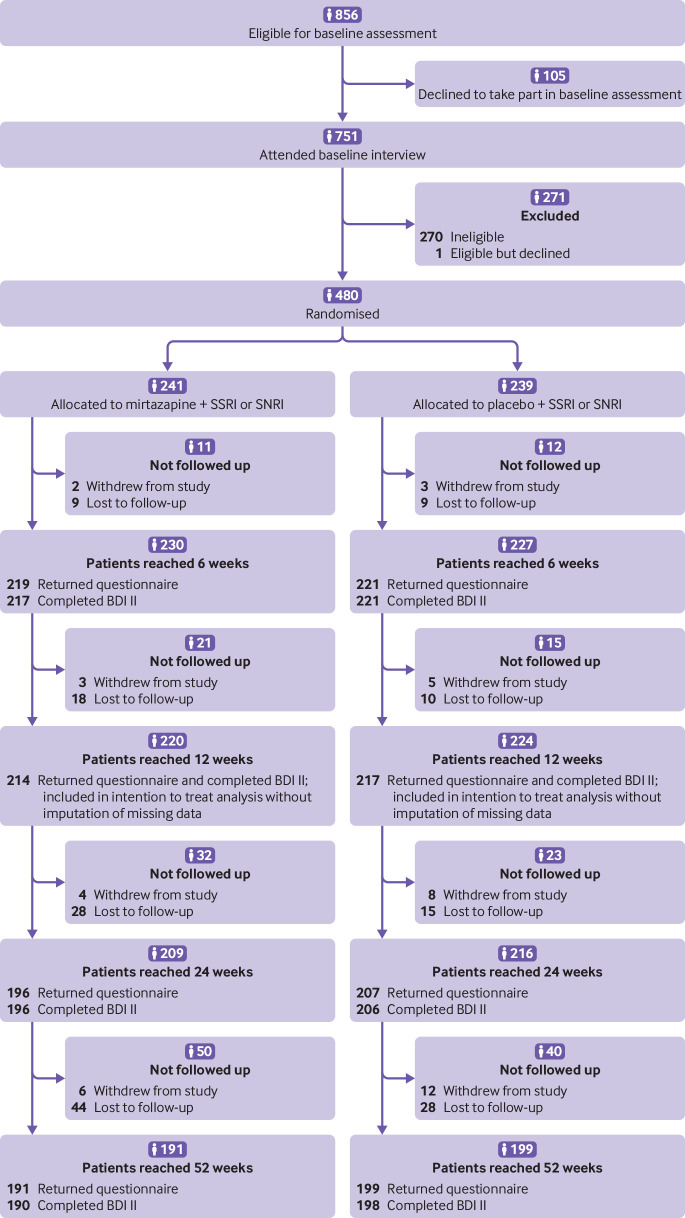
Flow of participants through study. SSRI=selective serotonin reuptake inhibitor; SNRI=serotonin-noradrenaline reuptake inhibitor; BDI II=Beck depression inventory, second revision

The screening process started on 1 August 2013, and the final participant was randomised to the trial on 6 October 2015. The follow-up data were collected between August 2015 and the end of October 2016. Of 856 patients identified as potentially eligible and invited to attend a baseline appointment, 105 (12%) declined. Those who declined were comparable to attenders on age, sex, and home ownership but were less likely to be educated to A level or above (31% *v* 48%). At baseline, one patient was eligible but declined, one was alcohol dependent, one had recently had the dose of antidepressant altered, and 268 did not satisfy the ICD-10 criteria for a major depressive episode or had a BDI II score less than 14, or both. A total of 480 participants were randomised (mirtazapine and SSRI or SNRI: n=241; placebo and SSRI or SNRI n=239); 431 (90%) were followed up at 12 weeks, 403 (84%) at 24 weeks, and 390 (81%) at 52 weeks (fig 1).

The two groups had similar baseline characteristics, but some evidence showed that participants in the mirtazapine group had more severe depression (table 1). Participants randomised to mirtazapine were more likely to have a history of depression, and a higher proportion had had suicidal thoughts in the past.

**Table 1:** Baseline characteristics of randomised participants. Values are numbers (percentages) unless stated otherwise

Characteristics	Allocated groups		
	Mirtazapine+SSRI or SNRI (n=241)	Placebo+SSRI or SNRI (n=239)	
**Stratification variable**			
Site:			
Bristol	89 (37)	88 (37)	
Exeter	61 (25)	61 (25)	
Keele/North Staffs	41 (17)	41 (17)	
Hull	50 (21)	49 (21)	
**Minimisation variables**			
Women	168 (70)	164 (69)	
Baseline BDI II score:			
14-25	77 (32)	79 (33)	
26-34	78 (32)	78 (33)	
≥35	86 (36)	82 (34)	
Currently receiving psychological services	33 (14)	29 (12)	
**Sociodemographic variables**			
Mean (SD) age (years)	50.4 (13.8)	49.9 (12.5)	
Ethnic group:			
White	233 (97)	235 (98)	
Non-white	8 (2)	4 (2)	
Marital status:			
Married or cohabiting	142 (59)	135 (56)	
Single	47 (20)	53 (22)	
Separated, divorced, or widowed	52 (22)	51 (21)	
Employment status:			
Not working	132 (55)	104 (44)	
Educational attainment:			
A level or higher	115 (48)	115 (48)	
GSCE, standard grade, or O level or equivalent	72 (30)	78 (33)	
No formal qualification	54 (22)	46 (19)	
Financial wellbeing:			
Just about getting by or worse	130 (54)	126 (53)	
Median (interquartile range) alcohol use score*	2.0 (1.0-4.0)	2.0 (1.0-4.0)	
Mean (SD) No of life events in past six months	1.0 (1.0)	1.1 (1.0)	
Mean (SD) social support score	12.2 (4.1)	12.8 (4.0)	
**Caring responsibilities**			
Providing care for someone with a disability	30 (12)	37 (15)	
**Measures of depression**			
Previous depression	206 (85)	190 (79)	
Previous referral to psychiatrist for depression†:	71 (34)	60 (32)	
Previous episodes of depression‡:			
0	3 (1)	5 (3)
1	14 (7)	8 (4)
2-4	82 (40)	79 (42)
≥5	107 (52)	98 (52)
Length of current course of antidepressants (months):		
<6	26 (11)	20 (8)
≥6	215 (89)	219 (92)
ICD-10 primary diagnosis:		
Mild	38 (16)	44 (18)
Moderate	138 (57)	144 (60)
Severe	65 (27)	51 (21)
Mean (SD) scores:		
CIS-R	28.3 (8.2)	27.0 (8.3)
BDI II	31.5 (10.2)	30.6 (9.6)
GAD-7§	11.3 (4.8)	10.7 (4.8)
PHQ-9	16.7 (5.5)	16.0 (5.5)
EQ-5D-5L¶	0.65 (0.26)	0.69 (0.22)
SF-12 aggregate physical functioning**	45.7 (13.8)	46.4 (13.1)
SF-12 aggregate mental functioning**	27.9 (9.6)	29.2 (9.7)
Suicidal ideation:		
None	81 (34)	119 (50)
Patient feels life isn’t worth living	59 (24)	44 (18)
Suicidal thoughts/plans	101 (42)	76 (32)

SSRI=selective serotonin reuptake inhibitor; SNRI=serotonin-noradrenaline reuptake inhibitor; BDI II=Beck depression inventory, second revision; ICD-10=international classification of diseases, 10th revision; CIS-R=revised clinical interview schedule; GAD=generalised anxiety disorder; PHQ=patient health questionnaire.

* AUDIT score.^26^

Number of missing observations by treatment group: †n=35 mirtazapine, n=49 placebo; ‡n=35 mirtazapine, n=49 placebo; §n=3 mirtazapine, n=0 placebo; ¶n=1 mirtazapine, n=1 placebo; **n=7 mirtazapine, n=4 placebo.

At 12 weeks, the mean BDI II score in those randomised to the usual care and mirtazapine group was 18.0 (SD 12.3) compared with 19.7 (12.4) in those randomised to usual care and placebo (table 2). A small difference in favour of the intervention was found after adjustment for baseline BDI II score and the stratification and minimisation variables, site, baseline thirds of BDI II score, sex, and whether the participant was receiving psychological therapy at baseline. The confidence interval included the null; it is therefore possible that the two treatment groups did not differ (adjusted difference in means −1.83, 95% confidence interval −3.92 to 0.27, P=0.09); table 2). Slightly larger differences were observed in a per protocol and complier average causal effect analyses (see supplementary table A1). Further adjustment for characteristics showing an imbalance at baseline did not materially affect the results of the primary analysis (see supplementary table A2).

**Table 2:** Beck depression inventory, second revision (BDI II) scores between treatment groups at baseline and 12, 24, and 52 weeks

Variables	Mirtazapine+SSRI or SNRI			Placebo+SSRI or SNRI			Comparison		
	No	Mean (SD)		No	Mean (SD)		Adjusted* difference in means (95% CI)	P value	Effect size (Cohen’s d)
Baseline	241	31.5 (10.2)		239	30.6 (9.6)		-	-	-
**Primary outcome**									
12 weeks	214	18.0 (12.3)		217	19.7 (12.4)		−1.83 (−3.92 to 0.27)	0.09	0.148
**Secondary outcomes**									
24 weeks	196	17.3 (12.9)		206	18.2 (12.6)		−0.85 (−3.12 to 1.43)	0.46	0.066
52 weeks	190	16.8 (12.7)		198	16.7 (12.2)		0.17 (−2.13 to 2.46)	0.89	0.014

SSRI=selective serotonin reuptake inhibitor; SNRI=serotonin-noradrenaline reuptake inhibitor.

* Adjusted for baseline BDI II score and stratification and other minimisation variables.

At 24 and 52 weeks, the adjusted difference in BDI II score between the two groups was smaller and included the null (24 weeks: adjusted difference in means −0.85 (−3.12 to 1.43); 52 weeks: adjusted difference in means 0.17 (−2.13 to 2.46); table 2). Adopting per protocol and complier average causal effect approaches to analysis of these outcomes yielded similar or slightly larger differences (see supplementary table A1).

Participants were able to request unblinding after the primary outcome at 12 weeks. The results in table 2 at 24 and 52 weeks include all those who remained in the trial, unblinded or not. Eighty three participants in the mirtazapine group and 103 in the placebo group requested unblinding by 52 weeks. A sensitivity analysis at 24 and 52 weeks found no between group differences in BDI II score among those who remained blinded throughout the trial (see supplementary table A3).

The between group differences in all the secondary outcome scores at 12 weeks were in favour of the intervention, including a second measure of depressive symptoms, the patient health questionnaire-9. However, the differences were small, and in almost every case (apart from the GAD-7, which measures anxiety symptoms, and the mental health component of the SF-12) the confidence interval for the difference included the null (table 3). Adherence to the trial drug was substantially lower in the intervention group compared with placebo group (table 3). Outcomes at later time points showed smaller between group differences (see supplementary table A4).

**Table 3:** Secondary outcomes at 12 weeks

Outcomes	Mirtazapine+SSRI or SNRI				Placebo+SSRI or SNRI				Comparison		
	No	No (%)	Mean (SD)		No	No (%)	Mean (SD)		Adjusted odds ratio* (95% CI)	Adjusted* difference in means (95% CI)	P value
Response	214	94 (44)	-		217	78 (36)	-		1.39 (0.94 to 2.07)	-	0.10
Remission	214	63 (29)	-		217	53 (24)	-		1.29 (0.82 to 2.02)	-	0.27
GAD-7	214	-	7.15 (5.63)		217	-	7.89 (5.78)		-	−0.98 (−1.93 to −0.03)	0.04
EQ-5D-5L	213	-	0.72 (0.27)		216	-	0.73 (0.25)		-	0.01 (−0.02 to 0.05)	0.40
SF-12 (physical)	208	-	44.09 (12.87)		210	-	45.85 (12.54)		-	−1.09 (−2.75 to 0.57)	0.20
SF-12 (mental)	208	-	39.94 (12.27)		210	-	36.33 (12.53)		-	3.91 (1.63 to 6.20)	0.001
PHQ-9	212	-	9.74 (6.35)		217	-	10.63 (6.21)		-	−1.05 (−2.14 to 0.04)	0.06
Adherence	210	156 (74.3)	-		214	180 (84.1)	-		0.55 (0.34 to 0.89)	-	0.01
ASEC	184	-	10.13 (7.02)		206	-	9.77 (7.93)		-	0.35 (−1.04 to 1.73)	0.62

SSRI=selective serotonin reuptake inhibitor; SNRI=serotonin-noradrenaline reuptake inhibitor; GAD-7=generalised anxiety disorder-7; SF-12=short form 12; PHQ-9=patient health questionnaire-9; ASEC=antidepressant side effect checklist.

* Adjusted for baseline values of outcome and stratification and minimisation variables except in the case of adherence at 12 weeks where adjustment was made only for stratification and minimisation variables.

No between group difference was found for adverse effects using the antidepressant side effect checklist at 12 weeks (table 3). We also collected spontaneous participant reports of adverse effects. In the first 12 weeks most reported adverse effects were minor. Eleven serious adverse events resulted in hospital admission, of which eight occurred in the intervention group (see supplementary table A5). More patients in the intervention group reported non-serious adverse effects, and 46 participants reporting adverse effects in this group stopped their drug compared with nine in the placebo group (table 4).

**Table 4:** Most common types of adverse events (AEs) spontaneously reported by participants at 12 weeks from randomisation*

System affected, with examples of AEs	Mirtazapine+SSRI or SNRI (n=241)			Placebo+SSRI or SNRI (n=239)	
	No (%) of patients reporting AE	No of patients reporting AE who stopped study drug		No (%) of patients reporting AE	No of patients reporting AE who stopped study drug
Anticholinergic:					
Dry mouth, blurred vision, urinary difficulties	16 (7)	3		4 (2)	0
Central nervous system:					
Drowsy, light headed, headache, unpleasant dreams	59 (24)	23		20 (8)	2
Increase in appetite or weight gain	26 (11)	7		8 (3)	0
Psychiatric:					
Increase in anxiety	8 (3)	4		5 (2)	0
Other:					
Restless legs, nausea, peripheral oedema	47 (20)	13		47 (20)	8
Any	121 (50)	46		71 (30)	9

SSRI=selective serotonin reuptake inhibitor; SNRI=serotonin-noradrenaline reuptake inhibitor.

* Patients may have reported more than one type of adverse event therefore column totals are greater than the total number of individuals reporting adverse effects.

We compared our analyses of the primary outcome using complete cases with analyses that tackled missing data. The findings using complete cases seemed to be robust to various assumptions about missing data (see supplementary table A6).

We found no evidence that either of the two preplanned subgroup analyses had any effect on the difference between the mirtazapine and placebo groups (P=0.101 for interaction with treatment group for baseline depression severity: P=0.30 for interaction with treatment group for treatment resistance).

## Discussion

This study did not find convincing evidence of a clinically important benefit for mirtazapine over placebo when given in addition to an SSRI or SNRI antidepressant for patients who had remained depressed after at least six weeks of antidepressant treatment, recruited from primary care.

In the primary analysis at 12 weeks, the placebo group improved from a baseline Beck depression inventory, second revision (BDI II) score of 30.6 to a mean of 19.7 and the intervention group from a baseline BDI II score of 31.5 to a mean of 18.0. We based our sample size calculation on detecting a between group difference equivalent to 3 or 4 BDI II points, which we considered would be clinically important. The adjusted difference (in means) between the groups after 12 weeks was less than this, at −1.83 (95% confidence interval −3.92 to 0.27, P=0.09) points on the BDI II in favour of the intervention group. Although the lower limit of the 95% confidence interval for this difference includes the possibility of a clinically meaningful effect, the confidence interval also includes the null and the most likely (mean) effect is small, making clinical benefit unlikely.

Similar observations of small differences between the treatment groups in favour of the mirtazapine group were observed for the secondary outcomes at 12 weeks, but for most outcomes the 95% confidence intervals surrounding the difference between groups included the null. This weak evidence of a small effect at 12 weeks is supported by changes in favour of the intervention group in the SF-12 aggregate mental health score (between group difference 3.91, 95% confidence interval 1.63 to 6.20) and generalised anxiety disorder-7 (−0.98, −1.93 to −0.03) where confidence intervals did not include the null, although the clinical importance of these small differences is not clear. Outcomes at later time points showed smaller between group differences with no evidence of benefit over the longer term. Complier average causal effect and per protocol analyses for the primary outcome, designed to estimate treatment effects in those who complied with their allocated treatment, showed slightly larger between group differences than the primary analyses, but these were still consistent with a chance observation, and per protocol analyses are known to be biased. Prespecified subgroup analyses based on severity and degree of treatment resistance did not yield any evidence of effect modification.

In the mirtazapine group, 46 participants who reported adverse effects stopped their drug compared with nine in the placebo group. Adherence was therefore substantially lower in the mirtazapine group than placebo group and is likely to have been a consequence of adverse effects. Although the two groups did not differ in their rating of adverse effects using the antidepressant side effect checklist scale, this may be in part due to the lower rate of adherence to the trial drug in the intervention group. The number of serious adverse events was small in both groups, and none were directly attributable to the intervention.

### Strengths and weaknesses of this study

Participants, investigators, and assessors were blind to the allocation up to and including the primary outcome at 12 weeks. Follow-up rates throughout the trial were good at all sites, with overall follow-up rates of 90% at 12 weeks, 84% at 24 weeks, and 81% at 52 weeks. Sensitivity analyses were done to assess the impact of missing data on the analysis of the primary outcome. Whether the missing data were estimated under the assumption of a best or worst case scenario or using multiple imputation, the observed difference in BDI II scores at 12 weeks between treatment groups was small. Some minor baseline imbalances existed between the two groups but adjustment for these did not materially affect the results.

The criteria for defining inadequate response to treatment that we adopted have been used elsewhere in primary care research^20^ and were designed to be inclusive while reflecting treatment guidelines from the National Institute for Health and Care Excellence.^4^ Our approach accords with the Maudsley staging method, where treatment failure after an adequate dose of an antidepressant for six weeks is an important starting point on a continuum of treatment failure.^27^ The authors point out that in addition to treatment failure, severity and duration of depression are important dimensions of treatment resistance. Nearly all (90%) of our participants had been taking an antidepressant for at least six months, and the range of symptom severity in our sample was evenly spread between mild to moderate, moderate to severe, and severe groups. In addition, most participants reported previous episodes of depression. Hence the population recruited to the study is representative of the group for whom there is uncertainty around ongoing management in primary care.

We based our view of the minimal clinically important difference between intervention and placebo groups of 3 or 4 points in BDI II score on previous recommendations from NICE.^4^ Since the writing of our protocol an approach towards establishing minimal clinically important difference using self rated global ratings of improvement has been developed.^14^ This approach gives an estimate of a minimal clinically important difference in depression of 17.5% reduction in BDI II score for a depressed primary care population but suggests that the minimal clinically important difference is higher, at 32% in a non-responsive population similar to that studied here. This translates to differences of 3.5 and 5.9 BDI II points, respectively. It therefore seems unlikely that mirtazapine would provide a clinically important benefit, although there is still considerable uncertainty around the clinically important difference in treatment outcome for this group of patients.

### Comparison to other studies

Two earlier small studies, one of which was in treatment resistant patients^8^ and one in those who had responded to previous treatment^9^, reported that mirtazapine in combination with an SSRI gave a greater improvement than monotherapy. A further recent study also reported benefit in non-resistant patients and that mirtazapine was well tolerated in combination with either an SSRI or venlafaxine (an SNRI).^10^ The STAR*D study^3^ compared venlafaxine plus mirtazapine with tranylcypromine, a monoamine oxidase inhibitor antidepressant. Although the combination of venlafaxine and mirtazapine showed a modest advantage over the monoamine oxidase inhibitor, no placebo group was included in this comparison. The large CO-MED (Combining Medications to Enhance Depression Outcomes) randomised trial compared the combination of venlafaxine and mirtazapine with escitalopram (an SSRI) and placebo in patients who had either recurrent depression or chronic depression lasting at least two years.^11^ Response rates did not differ between the two groups, but the burden of adverse effects was greater in the combined antidepressant group. Those recruited into CO-MED differed from our study population in that they were not necessarily taking an antidepressant at baseline.

### Unanswered questions

Half of those who take antidepressants in an adequate dose for an adequate duration remain depressed.^3^ ^28^ This represents a substantial burden of illness and an unmet or inadequately met need. Although many patients in this group can benefit from cognitive behavioural therapy, it is not always easily available nor is it universally effective.^20^ In primary care, where most initial encounters between people with depression and clinicians take place, antidepressants are still widely prescribed and remain a first line treatment. Several drug strategies have been developed to help those who do not respond to first line treatment, but the evidence supporting them is not of high quality.^6^ There is therefore a lack of clear guidance for clinicians in an area of unmet need, and this is particularly important in primary care because of the size of the population who experience no improvements from antidepressant treatment.^28^

### Conclusion

The lack of clear evidence of benefit in our study, combined with the increased burden of adverse effects in the mirtazapine group, means that we cannot recommend this combination as a routine strategy in primary care for those who remain depressed after adequate treatment with SSRI or SNRI antidepressants.

Box start

## What is already know on this topic

Half of those in primary care who take antidepressants remain depressed despite adhering to treatment
There is a pharmacological rationale for adding mirtazapine, an antidepressant with a different and complementary mode of action, to the widely prescribed selective serotonin reuptake inhibitor (SSRI) and serotonin and noradrenaline reuptake inhibitor (SNRI) antidepressants—evidence from several small studies suggests that this combination might be effective
It was important to study this in primary care where most depression is diagnosed and managed, and this combination is used with increasing frequency

## What this study adds

This study did not find evidence of a clinically important benefit for mirtazapine in addition to an SSRI or SNRI over placebo in primary care patients with treatment resistant depression
Those who took mirtazapine were more likely to experience adverse effects and to stop treatment
These findings challenge the growing practice of the addition of mirtazapine to SSRI or SNRI in this group of patients

## Web extra

Extra material supplied by authors

**Supplementary information:** tables A1-A6

## Notes

Contributors: DSK, GL, SD, NJW, TJP, WH, IMA, JC, CMD, CAC-G, and UM were responsible for the original proposal and securing funding for the trial. DT joined them in drafting the original protocol. DSK as chief investigator had overall responsibility for the management of the study and the Bristol site. JC and CMD had responsibility for the Exeter site, UM for the Hull site, and CAC-G for the Keele/North Staffs site. All authors contributed to the refinement of the protocol. DT and AB had overall responsibility for the data collection and were supported by CJ, MC, TS, and HG. SJM, TJP, and NJW wrote the statistical analysis plan. SJM did the main analyses with input from TJP, NJW, and DSK. DSK wrote the initial draft of the report. He is the guarantor. All authors contributed to and approved the final report. The corresponding author attests that all listed authors meet authorship criteria and that no others meeting the criteria have been omitted.

Ethical approval: This study was approved by the South East Wales research ethics committee C (ref 12/WA/0350). Participants gave informed consent to take part in the study.

Data sharing: Individual participant data that underlie the results reported in this article, after deidentification (text, tables, figures, and appendices) will be available beginning three months and ending five years after article publication to researchers who provide a methodologically sound proposal. The study protocol and statistical analysis plan will also be available. To gain access, data requestors will need to sign a data access agreement. Proposals should be directed to david.kessler@bristol.ac.uk.

Transparency: The lead author affirms that the manuscript is an honest, accurate and transparent account of the study and that no important aspects of the study have been omitted.
