# Supplementary material for: Mirtazapine added to SSRIs or SNRIs for treatment resistant depression in primary care: phase III randomised placebo controlled trial (MIR)
Source: BMJ. 2018 Oct 31;363:k4218. doi: 10.1136/bmj.k4218 (PMC6207929; doi:10.1136/bmj.k4218)
Supplement: Supplementary file 1 — Supplementary information: tables A1-A6 [file kesd045206.ww1.pdf]

Appendix

Table A1 Per protocol and CACE analyses of BDI-II scores

	Number of patients in model	Difference in means*		95% CI	p-value
	**CACE**				
**12 weeks**	427	-2.39		-5.18 to 0.40	0.09
**24 weeks**	396	-1.02		-3.04 to 1.90	0.49
**12 months**	379	0.17		-2.91 to 3.25	0.91
	**Per protocol**				
**12 weeks**	327	-2.18		-4.60 to 0.24	0.08
**12 months**	138	-1.08		-5.11 to 2.95	0.60

* Adjusted for baseline BDI-II score, stratification and minimisation variables

Table A2: Means and differences in mean BDI-II scores at 12 weeks, 24 weeks and 12 months adjusting for baseline BDI-II scores, stratification and other minimisation variables, history of depression, length of current course of antidepressants and suicidal ideation

	**Mirtazapine + SSRI/SNRI**		**Placebo + SSRI/SNRI**		**Comparison**	
	N	*Mean (SD)*	*N*	*Mean (SD)*	*Adjusted difference in means (95% CI)*	*p-value*
Primary outcome						
12 weeks	*214*	*18.0 (12.3)*	*217*	*19.7 (12.4)*	*-2.12 (-4.25 to 0.02)*	*0.05*
Secondary outcomes						
24 weeks	*196*	*17.3 (12.9)*	*206*	*18.2 (12.6)*	*-1.26 (-3.57 to 1.05)*	*0.28*
12 months	*190*	*16.8 (12.7)*	*198*	*16.7 (12.2)*	*-0.29 (-2.61 to 2.03)*	*0.81*

*Table A3: Means and difference in mean BDI-II scores between treatment groups at 24 and 52 weeks among those remaining blinded**

	**Mirtazapine + SSRI/SNRI**		**Placebo + SSRI/SNRI**		**Comparison**	
	N	*Mean (SD)*	*N*	*Mean (SD)*	*Adjusted** difference in means (95% CI)*	*p-value*
*24 weeks ****	*152*	*17.4 (13.0)*	*141*	*17.0 (12.7)*	*0.26 (-2.41 to 2.94)*	*0.85*
*52 weeks ****	*120*	*16.5 (13.1)*	*110*	*15.1 (11.4)*	*1.48 (-1.57 to 4.53)*	*0.34*

**This is a post-hoc exploratory analysis and was not part of the original SAP*

***: adjusted for baseline BDI-II score and the stratification and other minimisation variables*

****: One patient in the placebo group returned a 24-week questionnaire without a BDI-II measure as did one patient in the mirtazapine group at 52 weeks*

Table A4: Secondary outcomes at 24 weeks and 12 months (except BDI-II)

	**Mirtazapine + SSRI/SNRI**			**Placebo + SSRI/SNRI**			**Comparison**		
	N	N (%)	Mean (SD)	N	N (%)	Mean (SD)	Adjusted OR* (95% CI)	Adjusted* difference in means (95% CI)	p-value
24 weeks									
"Response"	196	96 (49.0)	-	206	100 (48.5)	-	1.01 (0.67 to 1.50)	-	0.98
"Remission"	196	65 (33.2)	-	206	59 (28.6)	-	1.28 (0.81 to 2.01)	-	0.29
GAD-7	195	-	6.83 (5.89)	206	-	7.17 (5.86)	-	-0.56 (-1.56 to 0.44)	0.27
EQ-5D-5L	196	-	0.72 (0.25)	207	-	0.74 (0.25)	-	0.01 (-0.02 to 0.05)	0.46
SF-12 (physical)	191	-	42.88 (13.02)	201	-	45.37 (12.75)	-	-1.54 (-3.23 to 0.15)	0.07
SF-12 (mental)	191	-	39.89 (13.92)	201	-	37.91 (12.43)	-	2.32 (-0.17 to 4.80)	0.07
12 months									
"Response"	190	97 (51.1)	-	198	101 (51.0)	-	0.99 (0.66 to 1.49)	-	0.98
"Remission"	190	63 (33.2)	-	198	67 (33.8)	-	0.96 (0.62 to 1.50)	-	0.87
GAD-7	189	-	6.81 (6.23)	198	-	6.80 (5.73)	-	-0.17 (-1.23 to 0.90)	0.75
EQ-5D-5L	189	-	0.72 (0.28)	199	-	0.75 (0.25)	-	0.001 (-0.04 to 0.04)	0.95
SF-12 (physical)	182	-	43.34 (13.42)	191	-	44.32 (12.49)	-	-0.47 (-2.19 to 1.24)	0.59
SF-12 (mental)	182	-	40.54 (13.80)	191	-	39.25 (13.09)	-	1.42 (-1.20 to 4.04)	0.29

ASEC	119		9.50 (7.65)	136	-	9.59 (8.26)	-	-0.43 (-2.19 to 1.33)	0.63

* Adjusted for baseline values of the outcome and stratification and minimisation variables except in the case of adherence at 12 weeks where adjustment was made solely for stratification and minimisation variables

Table A5 Serious Adverse Events in the 12 weeks up to the primary outcome (all requiring hospitalisation).

**Allocation**	**Brief description of event**	**Relatedness to IMP as rated at follow-up**
Mirtazapine	Fall leading to minor injury, observed overnight	Not related
Mirtazapine	Admitted to hospital as a day case for pre-planned gynaecological procedure (D&C).	Not related
Mirtazapine	Deep Vein Thrombosis	Not related
Mirtazapine	Transient Ischaemic Attack	Unlikely to be related
Mirtazapine	Dental Extraction	Not related
Mirtazapine	Suicidal ideation and self-harm	Possibly related
Mirtazapine	Deliberate Overdose	Possibly related
Mirtazapine	Pancreatitis (pre-existing gallstones)	Unlikely to be related
Placebo	Fall, broken rib. Had not started IMP.	Not related
Placebo	Infective gastroenteritis (norovirus)	Not related
Placebo	Fall leading to Ankle fracture	Not related

In the period following the primary outcome, between 12 and 52 weeks when participants could be voluntarily unblinded, there were 36 SAEs, 20 of which occurred in those allocated to the Mirtazapine group. None of these were attributable to the IMP.

Table A6: Comparison of results of primary analysis of complete cases with corresponding (ITT) analysis where missing data were imputed using "best" and "worst" case scenarios and multiple imputation for primary outcome of BDI-II score at 12 weeks

	**N**	**Difference in means***	**95% CI**	**p-value**
**Complete case**	431	-1.83	-3.92 to 0.27	0.09
**"Best" case scenario**	480	-2.22	-4.41 to -0.03	0.05
**"Worst" case scenario**	480	-1.11	-4.11 to 1.89	0.47
**Multiple imputation**	480	-1.78	-3.90 to 0.34	0.10

* *Adjusted for baseline BDI-II score, stratification and minimisation variables*
